# Comparative Transcriptional Profiling of Three Super-Hybrid Rice Combinations

**DOI:** 10.3390/ijms15033799

**Published:** 2014-03-03

**Authors:** Yonggang Peng, Gang Wei, Lei Zhang, Guozhen Liu, Xiaoli Wei, Zhen Zhu

**Affiliations:** 1 State Key Laboratory of Plant Genomics and National Center for Plant Gene Research, Institute of Genetics and Developmental Biology, Chinese Academy of Sciences, Beijing 100101, China; E-Mails: ygpeng@genetics.ac.cn (Y.P.); weigang2007@gmail.com (G.W.); lzhang@genetics.ac.cn (L.Z.); xlwei@genetics.ac.cn (X.W.); 2 State Key Laboratory of Genetic Engineering, Institute of Plant Biology, School of Life Sciences, Fudan University, Shanghai 200433, China; 3 Key Laboratory of Genome Sciences and Information, Beijing Institute of Genomics, Chinese Academy of Sciences, Beijing 101300, China; E-Mail: gzhliu@genomics.org.cn

**Keywords:** rice, transcriptional profiling, heterosis, photosynthesis, circadian clock

## Abstract

Utilization of heterosis has significantly increased rice yields. However, its mechanism remains unclear. In this study, comparative transcriptional profiles of three super-hybrid rice combinations, *LY2163*, *LY2186* and *LYP9*, at the flowering and filling stages, were created using rice whole-genome oligonucleotide microarray. The *LY2163*, *LY2186* and *LYP9* hybrids yielded 1193, 1630 and 1046 differentially expressed genes (DGs), accounting for 3.2%, 4.4% and 2.8% of the total number of genes (36,926), respectively, after using the *z*-test (*p* < 0.01). Functional category analysis showed that the DGs in each hybrid combination were mainly classified into the carbohydrate metabolism and energy metabolism categories. Further analysis of the metabolic pathways showed that DGs were significantly enriched in the carbon fixation pathway (*p* < 0.01) for all three combinations. Over 80% of the DGs were located in rice quantitative trait loci (QTLs) of the Gramene database, of which more than 90% were located in the yield related QTLs in all three combinations, which suggested that there was a correlation between DGs and rice heterosis. Pathway Studio analysis showed the presence of DGs in the circadian regulatory network of all three hybrid combinations, which suggested that the circadian clock had a role in rice heterosis. Our results provide information that can help to elucidate the molecular mechanism underlying rice heterosis.

## 1. Introduction

The superior performance of F_1_-hybrids over their parents is defined as heterosis/hybrid vigor. Heterosis has a significant role in improving crop productivity [1]. As a staple food crop, rice plays a vital role in feeding the growing population, and hybrid rice has contributed greatly to world-wide grain yields [2]. Rice is also a good model plant for studying heterosis, due to its small genome size, high collinearity with other Gramineae crops and abundant genomic data resources [3]. However, the molecular mechanism underlying heterosis in rice still remains elusive [4–6].

The traditional interpretation of heterosis can mainly be explained by three hypotheses: dominance hypothesis [7,8], over-dominance hypothesis [9,10] and epistasis [11]. All these hypotheses are not connected with molecular principles, and therefore, it is difficult to explain the molecular basis of heterosis [4,12]. Genetic dissection of quantitative trait variation indicated that multiple genes may be collectively responsible for the superior performance of F_1_-hybrids [13]. Meanwhile, a single heterozygous gene, *SINGLE FLOWER TRUSS*, was reported to cause hybrid vigor in hybrid tomato [14].

The superior performance of hybrids may result from the variations in expressed genes between F_1_-hybrids and their parents. Genome-wide gene expression analysis between hybrids and their parents has been performed extensively in rice [15–20], maize [21,22], wheat [23–25] and Arabidopsis [26], covering tissues, such as endosperm, roots and leaves, and various developmental stages. Several studies have found that a number of differentially expressed genes responsible for heterosis tend to be specific to different tissues and developmental stages [15,18,27,28] and that the genetic basis of heterosis is trait-dependent [29]. To date, there has been little consensus on this subject, as previous studies were performed separately with different samples, different strategies and different methods. In maize, there were attempts to study hybrid-parental gene expression among various hybrid combinations [30]. Most of these studies focused on one hybrid combination and provide few clues as to the molecular mechanism underlying different hybrid rice combinations.

Previously, we have investigated the transcriptional profile of super-hybrid rice and its parents by whole-genome oligonucleotide microarray [18] and by serial analysis of gene expression (SAGE) [16]. Microarray analysis of the *LYP9* hybrid combination revealed that significant numbers of DGs were enriched in the energy metabolism and transport categories. The research into transcriptional and physiological metabolic changes in the *LY2186* hybrid combination showed that increased carbon fixation and photosynthetic capacity play roles in hybrid vigor. These results from one hybrid combination are insufficient to explain heterosis, so in this study, we performed comparative transcriptomic analyses on three high-yielding hybrid rice combinations, *LY2163*, *LY2186* and *LYP9*, at the flowering (Fw) and filling (Fi) stages in an attempt to identify components of heterosis common amongst rice hybrids. The three hybrid combinations all had yield vigor, but there were differences in their trait components. *LYP9* had a high grain number, and *LY2163* and *LY2186* had high grain setting rates and grain weights (China Rice Data Center) [31].

## 2. Results and Discussion

### 2.1. Transcriptional Profiles of Three Super-Hybrid Rice Combinations

**Figure 1.**
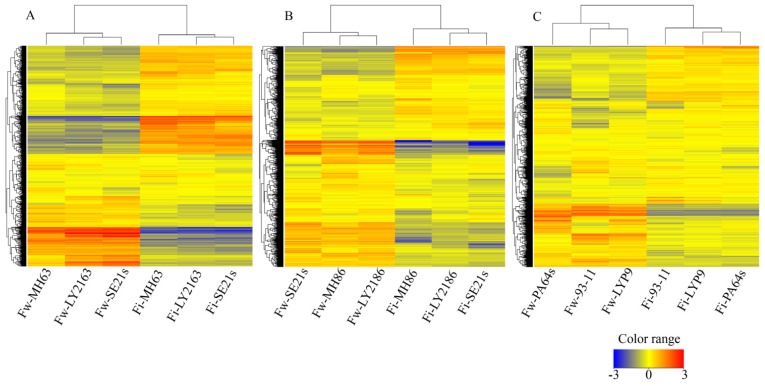
Clustering analysis of expressed genes among three hybrid combinations. Hierarchical clustering was done by GeneSpring 12.6 using normalized data. (**A**) *LY2163* hybrid combination; (**B**) *LY2186* hybrid combination; (**C**) *LYP9* hybrid combination. Fw, flowering; Fi, filling.

The gene expression profiles of flag leaves in three super-hybrid rice varieties, *Liangyou-2163* (*LY2163*), *Liangyou-2186* (*LY2186*) and *Liangyoupeijiu* (*LYP9*), and their parental lines (*LY2163* = *SE21s* × *Minghui63* (*MH63*), *LY2186* = *SE21s* × *Minghui86* (*MH86*) and *LYP9* = *PA64s* × *93–11*) were analyzed by rice whole-genome microarray. We detected 20,461 (*LY2163* hybrid combination), 31,516 (*LY2186* hybrid combination) and 19,950 (*LYP9* hybrid combination) expressed genes from the flowering stage and 16,977 (*LY2163* hybrid combination), 15,926 (*LY2186* hybrid combination) and 14,373 (*LYP9* hybrid combination) from the filling stage (Tables S1 and S2; Figure S1). Most of the expressed genes were shared by each hybrid combination. The identical genes accounted for 60.0%–73.5% of the expressed genes in each hybrid combination at the flowering stage and 75.6%–79.0% at the filling stage (Figure S1). Cluster analysis showed that the transcriptional profiles of the hybrids and their parents were different at each developmental stage and that similar expression patterns were observed among all three combinations at the same developmental stage (Figure 1). The expression patterns of all three hybrids were similar to their maternal parent at the filling stage. At the flowering stage, *LY2163* showed a similar expression to the maternal parent, but *LY2186* and *LYP9* were the opposite and resembled the paternal parent (Figure 1).

**Figure 2.**
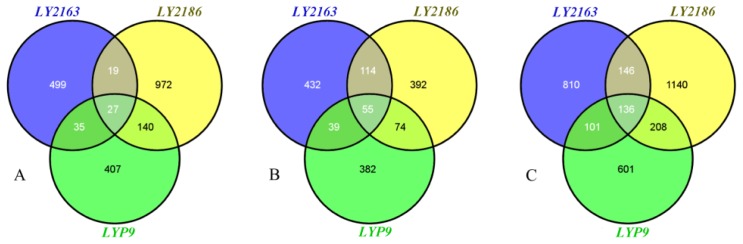
Venn diagram of differentially expressed genes (DGs) shared by three hybrid combinations. (**A**) DGs at the flowering (Fw) stage; (**B**) DGs at the filling (Fi) stage; (**C**) DGs at either the Fw or Fi stage.

**Figure 3.**
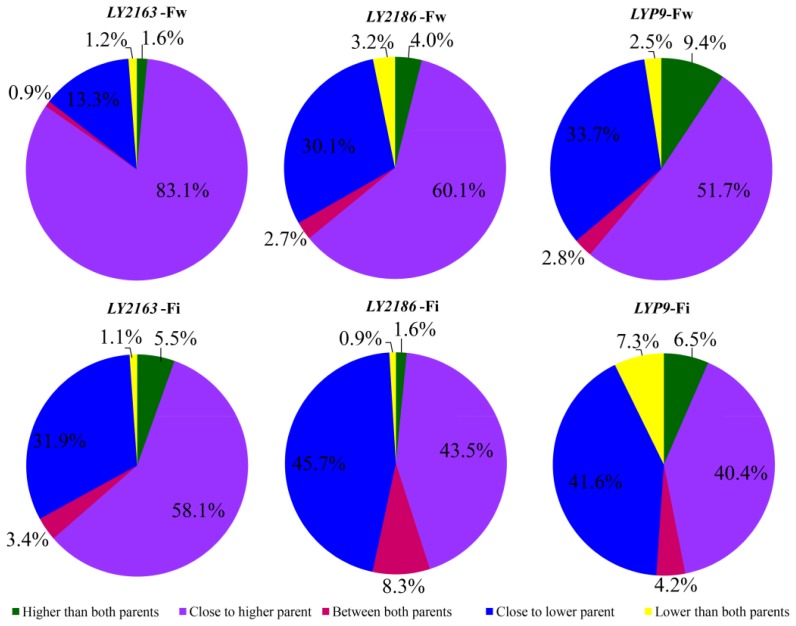
Pie graph representing expression patterns of DGs.

According to the statistical significance results for gene expression [18], we identified hundreds of differentially expressed genes (DGs) between the hybrid and its parents in each hybrid combination, unexpectedly, only a few DGs were shared by all three hybrid combinations (Figure 2A,B). To further find out how DG looks comparing to its two parents, the DGs of each hybrid combination at each stage were further classified into five expression patterns, higher than both parents (H2P), close to the higher parent (CHP), between both parents (B2P), close to the lower parent (CLP) and lower than both parents (L2P), based on the expression levels of these hybrid combinations. CHP and CLP made up the largest proportion and accounted for 82.0%–96.4% of the total DGs (Figure 3). The various gene expression patterns could result from the differential regulation of gene expression in the hybrid combinations [12,19].

As it is known that the changed gene expression between hybrid and parents is one of the important factors determining the phenotypic variation, DGs at either stage/tissue should both be involved in contributing to heterosis, though it is hard to determine specifically which genes contributed directly. To make the comparison more comprehensive, we also pooled up DGs of both stages in each hybrid combination while comparing differentially expressed genes among different combinations and found 1193, 1630 and 1046 DGs for the *LY2163*, *LY2186* and *LYP9* hybrid combinations, respectively (Figure 2C). These union DGs accounted for 3.2%, 4.4% and 2.8%, respectively, of the 36,926 genes studied (Table 1; Tables S3–S8); however, only 136 DGs were shared by all three combinations, which accounted for 8.3%–13.0% of the total DGs in each hybrid combination (Figure 2C).

### 2.2. DGs Functions in the Three Hybrid Combinations

**Figure 4.**
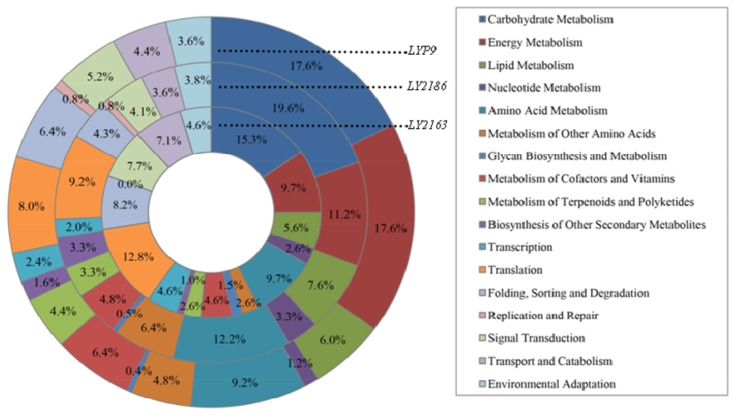
Graphical representation for the function classification of DGs in the F_1_ hybrid of three hybrid combinations.

Using the functional categories of the Kyoto Encyclopedia of Genes and Genomes (KEGG) pathway database by KEGG Orthology-Based Annotation System (KOBAS) [32], we classified 149 of the 1193 DGs in *LY2163*, 282 of 1630 DGs in *LY2186* and 185 of 1046 DGs in *LYP9* into 17 functional categories and found that all three hybrid combinations had a similar percentage of DGs in the classified functional categories (Figure 4). A large percentage of the DGs were involved in functional categories for carbohydrate metabolism and energy metabolism. A total of 15.3%, 19.6% and 17.6% of DGs of the *LY2163*, *LY2186* and *LYP9* hybrid combination, respectively, were classified into the carbohydrate metabolism category, and 9.7%, 11.2% and 17.6% of DGs, respectively, were classified into the energy metabolism category. These results were similar to those obtained in our previous study [16] and suggested that carbohydrate metabolism and energy metabolism may play roles in rice heterosis.

We used MicroArray Data Interface for Biological Annotation (MADIBA) to perform Gene Ontology (GO) analysis in order to investigate the functional categories of the genes further. The results showed that 254 out of 1193, 440 out of 1630, and 297 out of 1046 DGs at the flowering and filling stages were assigned to 87, 126 and 91 GO terms from the cellular component category, in *LY2163*, *LY2186* and *LYP9*, respectively (Table S9). In total, 39 GO terms are over-represented (*p* < 0.05) in the three combinations and two GO terms: the chloroplast and protein serine/threonine phosphatase complex were shared by three hybrid combinations. It is very interesting that among these GO terms, the DGs were significantly enriched in the chloroplast or chloroplast-related GO terms. DGs enrichment in the chloroplast indicates major changes in the photosynthetic ability of the F_1_ hybrids compared to their parents.

### 2.3. Metabolic Pathway Analysis of the DGs

The metabolic pathway analysis of total DGs by MADIBA [33] showed the involvement of 116 out of 1193 DGs in *LY2163*, 262 out of 1630 DGs in *LY2186* and 148 out of 1046 DGs in *LYP9* at either the flowering stage or filling stage (Table S10). Fisher’s exact test showed that the DGs were distributed in 71, 97 and 86, respectively, out of 143 metabolic pathways and were mainly enriched in categories, such as flavonoid biosynthesis and photosynthesis (light reactions). The carbon fixation pathway in particular showed significant DGs enrichment (*p* < 0.01) in all three super-hybrid combinations (Table 2, Table S10). The carbon fixation pathway transforms CO_2_ into organic compounds and can be a growth-limiting factor in modern agriculture [34]. The DGs enrichment of the carbon fixation pathway category suggested that carbon fixation efficiency may be enhanced in hybrid rice. Our previous study revealed that F_1_ hybrids showed more than 10% higher net photosynthetic rates than their parents in different hybrid combinations [16]. The results of the present study are consistent with previous findings that increased photosynthetic activity in the hybrids is responsible for heterosis [16,35].

We observed that some DGs belonging to certain metabolic pathways were shared in the three hybrid combinations (Figures S2–S4), e.g., genes encoding phosphoenolpyruvate carboxykinase (Os03g15050), pyruvate, phosphate dikinase (Os05g33570), phosphoribulokinase (Os02g47020) and fructose-bisphosphatase (Os01g64660) in the carbon fixation pathway and β-fructofuranosidase (Os02g01590) and β-glucosidase (Os04g43390) in the starch and sucrose metabolic pathway. A significant proportion of these DGs may play important roles in heterosis (Figures S2–S4). Meanwhile, DGs were also found to be involved in the flavonoid biosynthesis pathway in the three hybrid combinations (Table 2). For example, the chalcone synthase gene (Os11g32650), involved in the flavonoid biosynthesis pathway, is upregulated in both *LY2186* and *LYP9* hybrid combinations (compared to mid-parents) at the filling stage. Flavonoids have a variety of roles, including stress tolerance, UV-B protection [36,37] and regulation of auxin transport [38]. They have also been implicated in *Arabidopsis* heterosis [39,40]. Therefore, the flavonoid biosynthesis pathway may play a fundamental role in plant heterosis in *Arabidopsis* and rice.

### 2.4. Regulatory Network Analysis of DGs in the Three Hybrids

**Figure 5.**
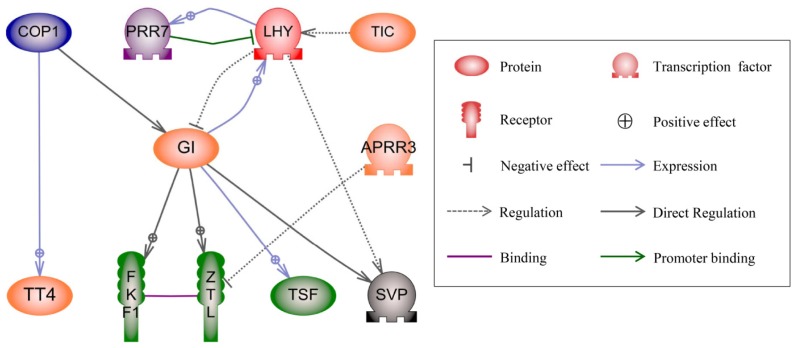
Gene regulatory network in three hybrid combinations. DGs in three hybrid combinations were used for direct interaction analysis by Pathway Studio 9.0. Interaction searching includes promoter binding, expression, regulation and binding. LHY, LATE ELONGATED HYPOCOTYL; PRR7, PSEUDO-RESPONSE REGULATOR 7; SVP, SHORT VEGETATIVE PHASE; TIC, TIME FOR COFFEE; GI, GIGANTEA; ZTL, ZEITLUPE; APRR3, PSEUDO-RESPONSE REGULATOR 3; FKF1, FLAVIN-BINDING, KELCH REPEAT, F-BOX 1; TSF, TWIN SISTER OF FT; COP1, CONSTITUTIVELY PHOTOMORPHOGENIC 1; TT4, TRANSPARENT TESTA 4. Black, dark green, navy refers to protein are encoded by DGs in *LY2163*, *LY2186* and *LYP9* respectively; dark magenta refers to protein are encoded by DGs in *LY2163* and *LYP9*; orange refers to protein are encoded by DGs in *LY2186* and *LYP9*; red refers to protein are encoded by DGs in *LY2163*, *LY2186* and *LYP9*.

Most genes usually need to operate synergistically with other genes to realize their biological functions [41]. To comprehensively understand the functions of DGs and their roles in heterosis, we constructed the DG-associated regulatory network in the three hybrid combinations. Eleven DGs involved in the circadian regulatory network were identified in the three hybrid combinations using Pathway Studio 9.0 software [42] (Table S11 and Figure 5). Of these 11 DGs, three DGs, *LATE ELONGATED HYPOCOTYL* (*LHY*), *PSEUDO-RESPONSE REGULATOR 7* (*PRR7*) and *SHORT VEGETATIVE PHASE* (*SVP*), were identified in the *LY2163* hybrid combination; eight DGs, *LHY*, *GIGANTEA* (*GI*), *ZEITLUPE* (*ZTL*), *TRANSPARENT TESTA 4* (*TT4*), *PSEUDO-RESPONSE REGULATOR 3* (*APRR3*), *TIME FOR COFFEE* (*TIC*), *FLAVIN-BINDING KELCH REPEAT F-BOX1* (*FKF1*) and *TWIN SISTER OF FT* (*TSF*), were identified in the *LY2186* hybrid combination; and seven DGs, *TIC*, *LHY*, *APRR3*, *PRR7*, *GI*, *CONSTITUTIVELY PHOTOMORPHOGENIC 1* (*COP1*) and *TT4*, were identified in the *LYP9* hybrid combination (Figure 5 and Table S11). In this network, the LHY activates the expression of *PRR7* [43], which, in turn, negatively regulates the expression of *LHY* [44] and forms a feedback loop (Figure 5). Another feedback loop is between GI and LHY, *GI* promotes the expression of *LHY* and *LHY* represses *GI* [45,46]. All these DGs encode key components of the circadian clock [47]. COP1 can modulate the light input signal to the circadian clock by targeting GI for degradation [48], and GI can interact with the blue light-sensing F-box proteins, FLAVIN-BINDING, KELCH REPEAT, F-BOX 1 (FKF1) [49] and *ZEITLUPE* (ZTL) [50], which are involved in regulating flowering time and circadian rhythms.

In this study, changes in the expression levels of circadian clock-related genes were identified in all three hybrids. The circadian clock can also coordinate plant physiological metabolism and responses to changes in the external environment to provide plants with a growth advantage [51]. The circadian clock can also regulate many biological processes, such as photosynthesis [52] and starch and sucrose metabolism [53]. Epigenetic modification of two key regulator genes (*CCA1* and *LHY*) in the circadian rhythm network resulted in altered levels of gene expression during photosynthesis and in the starch and sugar metabolic pathways, which led to growth vigor in Arabidopsis allopolyploids and hybrids [54]. Recent studies reported that methylation of *CCA1*, *LHY* genes and genes involved in flavonoid biosynthesis in hybrids, may be responsible for heterosis in Arabidopsis [40]. From the above, it is apparent that DGs involved in the circadian clock play a central role in rice heterosis.

### 2.5. Mapping of DGs Quantitative Trait Loci (QTLs)

**Figure 6.**
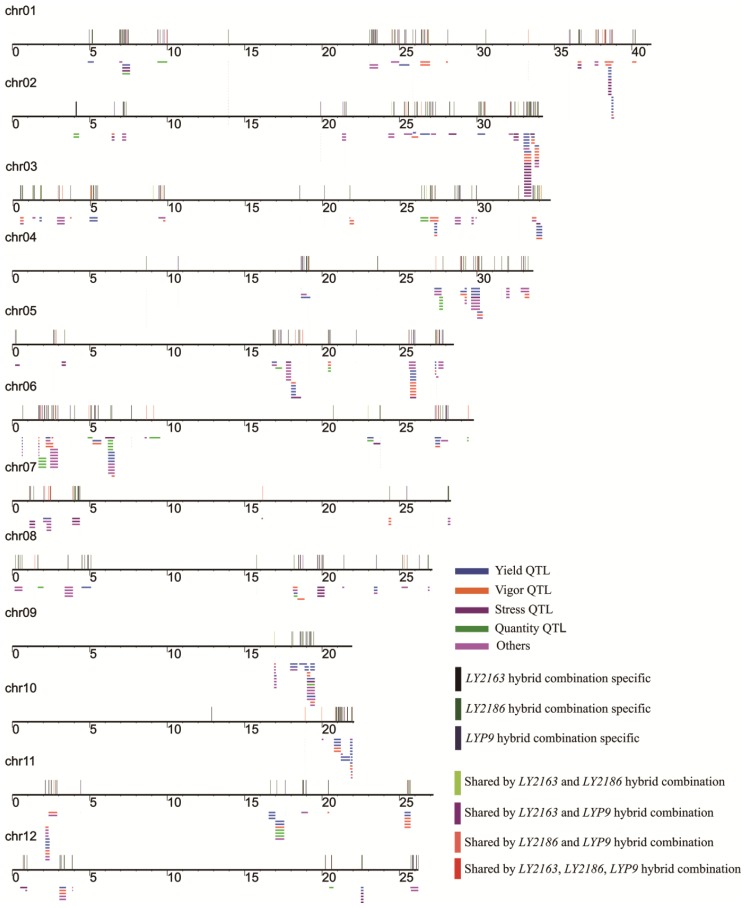
Distribution of DGs among QTLs of a small interval. QTLs in Gramene (number of harbored genes ≤ 100) and harbored DGs were aligned to the Michigan State University (MSU) Rice Genome Release 6.1. The long horizontal line represents 12 chromosomes of the rice genome; the short horizontal lines represent the QTL span, and short vertical lines represent DGs.

In order to examine whether DGs have potential associations with agronomic traits in hybrid rice, we mapped 2682 DGs to 4476 QTLs based on their genome coordinates in the Gramene database [55] (Table 3). Among them, 120 DGs and 3884 QTLs were shared by all three hybrid combinations. In the *LY2163*, *LY2186* and *LYP9* hybrid combinations; 989, 1385 and 884 DGs were mapped to 909, 1006 and 969 QTLs in the yield category, respectively (Table 3), and 882 QTLs were shared by all three hybrid combinations (Table 3). More than 80% of the DGs were located in QTLs, of which over 90% were in the yield-related QTLs in the three hybrid combinations. Similar results were found after the transcriptional profiling of the *LY2186* hybrid combination by Serial Analysis of Gene Expression (SAGE) [16]. Furthermore, in the *LY2163*, *LY2186* and *LYP9* hybrid combinations, we identified 164, 276 and 195 DGs that were located in 354, 591 and 424 QTLs, collectively 737 QTLs at small intervals of less than 100 genes (Figure 6).

Among the DG-located QTLs, many were well characterized, including panicle number (CQAQ9, CQJ1, CQK1, CQK2, *etc.*), seed number (CQAS138, CQAS142, CQAS21, CQAS25, *etc.*), 1000 seed weight (AQAK031, AQCY015, AQCY017, CQAS23, *etc.*) and grain yield (AQGK001, AQFF004, AQFF006, AQDK009, *etc.*). Functional annotation of some DGs can explain the QTL traits to some degree, for example soluble starch synthase 3 (Os04g53310) to AQCY010 for the filled grain number, glycoside hydrolase (Os03g03350) to CQAS39 for yield and malate dehydrogenase (Os01g46070) to CQN50 for the spikelet number. All the above results indicated that DGs can correlate with the superior heterotic traits in hybrid rice. Interestingly, the DG *GRAIN INCOMPLETE FILLING 1* (*GIF1*, Os04g33740) in the *LY2186* hybrid combination could be mapped to the yield-related QTLs involved in filled grain number, seed set percentage and 100 seed weight. A recent report showed that *GIF* could increase yield through improved grain filling [56].

As described above, although only a few shared DGs were found among the three hybrid combinations, they shared similar functional categories and mapped to the QTLs of similar agronomic traits, which indicated that in different hybrid combinations, genes that determined the traits were different, but they belonged to the same or similar pathways and played similar roles in determining the superior heterotic phenotype. Hybrids from different hybrid combinations may have different traits or the same traits may be regulated by different genes, because of their different breeding strategies and methods. However, the genes that determined the same traits may have been artificially selected, and the underlying molecular regulatory apparatus may be similar. These results suggested that different hybrid rice varieties in different combinations may have different genes contributing to the heterotic phenotype, but they all had a similar higher level regulatory mechanism. High grain yield is an important characteristic in all three hybrids and is determined mainly by three typical quantitative traits: the number of panicles, the number of grains per panicle and grain weight [57]. This suggests that the yield heterosis of the different combinations does not arise from a single or a small number of genes. Most of the DGs in the QTLs were different in the three tested hybrid combinations, but they tended to be located in similar QTLs. This suggested that the yield heterosis in the three hybrid combinations may be caused by different genes with more or less similar functions. In this study, we have established the potential correlations between DGs and agricultural traits and indicated promising insights for understanding the common mechanism of heterosis in rice.

## 3. Experimental Section

### 3.1. Plant Materials

Three super-hybrid rice cultivars: *LY2163* (*SE21s* × *MH63*), *LY2186* (*SE21s* × *MH86*) and *LYP9* (*PA64s* × *93-11*) were grown in the same field. The flag leaves of the field-grown plants at the flowering and filling stages were sampled, frozen in liquid nitrogen and stored at −80 °C.

### 3.2. Microarray Experiment and Data Processing

The whole-genome array was developed using the predicted genes of *indica 93-11* [58,59] with 70-mer oligonucleotides. The microarray was designed by the microarray laboratory at the Beijing Genomic Institute (Beijing, China) using results from previous studies [18,60], and the slides were processed according to the standard procedure [61]. Total RNA was extracted according to the published methods [62], quantified and labeled with Cy5 and Cy3 [63,64]. There were four biological replicates for each sample, and two dye swap hybridizations were carried out for each sample pair in order to reduce biological and technical errors. Separate tagged image file format images of the Cy5 and Cy3 channels were obtained by a ScanArray Lite scanner (Perkin-Elmer, Wellesley, MA, USA), and the spot intensities were quantified using Axon GenePix Pro 5.1 image analysis software (Axon Instruments, Foster City, CA, USA). The raw data was processed (excluding dirty points and bad points) according to the criteria described previously [18]. The processed data were normalized using the mean of all the expressed genes. The normalization of the two-channel data for each array was carried out using the intensity-based Loess method with R language (A language and environment for statistical computing). DGs were defined by a log-scale ratio of signal intensity between the hybrid and either of its parents with a *p* < 0.01 (*z*-test) [18].

### 3.3. Functional Category and Metabolic Pathway Analysis

For the expressed genes investigation, we performed functional annotations using Basic Local Alignment Search Tool (BLAST) [65,66] against the MSU Rice Genome Annotation Project Release 6.1. Cluster analysis of the expressed genes from the hybrid combinations was undertaken using the hierarchical clustering method and GeneSpring GX 12.6 software (Agilent Technologies, Palo Alto, CA, USA). Functional classification analysis of DGs was carried out by the KEGG orthology-based annotation system (KOBAS) version 2.0 [32]. The DGs pathway analysis was undertaken by MADIBA [33]. The *p*-value acquired was based on the number of enzymes found in each pathway by Fisher’s exact test.

### 3.4. Regulatory Network Analysis

The DGs list was analyzed for the presence of a regulatory network using Pathway Studio software (Ariadne Genomics, Rockville, MD, USA) [42], version 9.0, according to Entrez ID, which utilized DGs matched from the National Center for Biotechnology Information (NCBI) [67]. The ResNet Plant Database (version 4.0, Ariadne Genomics, Rockville, MD, USA) was used to identify the directed interactions among genes. These interactions were classified as promoter binding, expression, regulation and protein-protein binding types. False interaction relationships were eliminated using the references in the database.

### 3.5. Mapping DGs to Rice QTLs

Rice QTL data with physical positions on the MSU Rice Genome Annotation Project Release 6.1 were acquired from Gramene [55]. The DGs were mapped to rice QTLs using gene coordinates from the MSU Rice Genome Annotation Project, Release 6.1. QTLs at small intervals of less than 100 genes were mapped with DGs in the rice chromosomes, as described previously [18].

## 4. Conclusions

In this study, we analyzed the transcriptional profiles of three super-hybrid rice combinations at the flowering and grain filling stages. There were similar proportions of DGs in all three hybrid combinations; they had similar expression patterns and were mainly distributed in the carbohydrate metabolism and energy metabolism categories, which indicated that the three hybrid combinations had similar regulatory mechanisms. The DGs in each hybrid were particularly enriched in the carbon fixation pathway, the starch and sucrose metabolic pathway and the flavonoid biosynthesis pathway. The results were consistent with the higher photosynthesis and assimilation efficiency seen in hybrid rice [16]. We analyzed the DGs involved in the regulatory network and found that the circadian network could regulate DGs in the three important metabolic pathways mentioned above and played a key role in heterosis. Our research established a correlation between DGs and agricultural traits by mapping DGs to QTLs and revealed that a similar regulatory mechanism underlying heterosis may exist in a number of hybrid rice.

## Figures and Tables

**Table 1.** DGs identified at the flowering and filling developmental stages for the three hybrid rice combinations investigated.

Developmental stage	*LY2163*	*LY2186*	*LYP9*	Three hybrid combinations
Flowering (Fw)	580 (1.6%)	1,158 (3.1%)	609 (1.6%)	2,099 (5.7%)
Filling (Fi)	640 (1.7%)	635 (1.7%)	550 (1.5%)	1,488 (4.0%)
Fw + Fi	1,193 (3.2%)	1,630 (4.4%)	1,046 (2.8%)	3,142 (8.5%)

**Table 2.** The top ten DG-enriched metabolic pathways in the three hybrid rice combinations.

Overall rank a	Metabolic pathway b	*LY2163*		*LY2186*		*LYP9*	

		*p*-value c	Rank d	*p*-value c	Rank d	*p*-value c	Rank d
1	Carbon fixation	5.37 × 10^−3^	1	2.36 × 10^−6^	1	1.03 × 10^−4^	1
2	Flavonoid biosynthesis	0.185	3	0.192	4	0.116	3
3	Metabolism of xenobiotics by cytochrome P450	0.224	4	0.151	3	0.266	8
4	Photosynthesis	0.045	2	0.493	13	0.056	2
5	Reductive carboxylate cycle (CO_2_ fixation)	0.514	6	0.258	6	0.284	10
6	Oxidative phosphorylation	0.595	10	0.062	2	0.659	26
7	Glycolysis/Gluconeogenesis	0.779	17	0.414	10	0.345	11
8	Tricarboxylic acid (TCA) cycle	0.341	5	0.312	9	0.665	28
9	Starch and sucrose metabolism	0.755	16	0.297	8	0.632	23
10	Streptomycin biosynthesis	0.857	26	0.561	17	0.129	4

a The overall rank is rank of the average of the individual pathway ranks for the three hybrid combinations;

b metabolic pathway analysis was based on MicroArray Data Interface for Biological Annotation (MADIBA) [33];

c the *p*-value is provided by Fisher’s exact test; *p* < 0.05 was considered statistically significant;

d rank indicates the significance of the pathway in each hybrid combination.

**Table 3.** Correlations between DGs a and quantitative trait locus (QTL) b in the three hybrid rice combinations.

Category	*LY2163*	*LY2186*	*LYP9*	Shared by three hybrid combinations
Number of DGs	1,193	1,630	1,046	136
DGs located to QTL	1,004	1,411	894	120
DGs located to yield-related QTL	989	1385	884	119
Number of QTL located by DGs	4,057	4,326	4,114	3,884
QTL of yield category	909	1,006	969	882
QTL of vigor category	939	995	953	911
QTL of Sterility or fertility category	137	156	137	124
QTL of quality category	326	341	318	310
QTL of development category	427	450	421	412
QTL of abiotic stress category	346	365	339	315
QTL of biotic stress category	211	220	212	208
QTL of biochemical category	108	112	110	107
QTL of anatomy category	654	681	655	615

a Refers to differentially expressed genes between the hybrid and its parents;

b refers to QTLs in the Gramene database [55] that harbor DGs were aligned with the gene coordinates in Rice Genome Annotation Release 6.1.
